# [(1*S*,2*S*,3*R*,4*R*)-3-Hydr­oxy-4,7,7-tri­methyl­bicyclo­[2.2.1]heptan-2-yl]methyl[(*E*)-3-(trimethyl­silyl)prop-2-enyl]selen­onium bromide

**DOI:** 10.1107/S1600536808016863

**Published:** 2008-06-07

**Authors:** Hai-Yang Wang, Qiang Zhang, Yi-Zhi Li, Yuan Gui, Zhi-Zhen Huang

**Affiliations:** a School of Chemistry and Chemical Engineering, Nanjing University, Nanjing, 210093, People’s Republic of China; b State Key Laboratory of Coordination Chemistry, School of Chemistry and Chemical Engineering, Nanjing University, Nanjing, 210093, People’s Republic of China

## Abstract

The title compound, a seleno­nium bromide, C_17_H_33_OSeSi^+^·Br^−^, was obtained from the reaction of enanti­omerically pure 4,7,7-trimethyl-2-methyl­selanylbicyclo­[2.2.1]heptan-3-ol and (3-bromopropen­yl)trimethyl­silane in acetone. Due to the chiral bicyclic substituent, the crystal structure is not centrosymmetric and has no symmetry plane, with four chiral C atoms in the cation. The asymmetric unit contains one seleno­nium cation and one bromide anion. C–H⋯Br and O–H⋯Br hydrogen bonds link the ions, forming a one-dimensional *R*-helical chain-like supra­molecular structure.

## Related literature

For related literature, see: Li *et al.* (2005 ▶); Goodridge *et al.* (1988 ▶); Reich *et al.* (1975 ▶); Ye *et al.* (2002 ▶). 
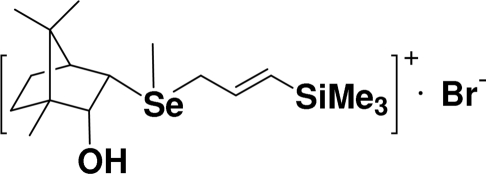


## Experimental

### 

#### Crystal data

C_17_H_33_OSeSi^+^·Br^−^
*M* *_r_* = 440.39
Monoclinic, 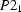

*a* = 7.555 (2) Å
*b* = 10.023 (2) Å
*c* = 14.423 (3) Å
β = 101.29 (3)°
*V* = 1071.0 (4) Å^3^
*Z* = 2
Mo *K*α radiation
μ = 3.67 mm^−1^
*T* = 291 (2) K
0.30 × 0.26 × 0.24 mm

#### Data collection

Bruker SMART APEX CCD diffractometer
Absorption correction: multi-scan (*SADABS*; Bruker, 2000 ▶) *T*_min_ = 0.35, *T*_max_ = 0.41
4460 measured reflections
3374 independent reflections
1732 reflections with *I* > 2σ(*I*)
*R*_int_ = 0.035

#### Refinement

*R*[*F* ^2^ > 2σ(*F* ^2^)] = 0.053
*wR*(*F* ^2^) = 0.102
*S* = 1.07
3374 reflections
170 parameters
1 restraint
H atoms treated by a mixture of independent and constrained refinement
Δρ_max_ = 0.64 e Å^−3^
Δρ_min_ = −0.74 e Å^−3^
Absolute structure: Flack (1983 ▶), 1140 Friedel pairs
Flack parameter: 0.01 (2)

### 

Data collection: *SMART* (Bruker, 2000 ▶); cell refinement: *SMART*; data reduction: *SAINT* (Bruker, 2000 ▶); program(s) used to solve structure: *SHELXTL* (Sheldrick, 2008 ▶); program(s) used to refine structure: *SHELXTL*; molecular graphics: *SHELXTL*; software used to prepare material for publication: *SHELXTL*.

## Supplementary Material

Crystal structure: contains datablocks global, I. DOI: 10.1107/S1600536808016863/im2070sup1.cif

Structure factors: contains datablocks I. DOI: 10.1107/S1600536808016863/im2070Isup2.hkl

Additional supplementary materials:  crystallographic information; 3D view; checkCIF report

## Figures and Tables

**Table 1:** Hydrogen-bond geometry (Å, °)

*D*—H⋯*A*	*D*—H	H⋯*A*	*D*⋯*A*	*D*—H⋯*A*
O1—H1*D*⋯Br1	0.87 (8)	2.28 (8)	3.143 (5)	175 (7)
C5—H5⋯Br1^i^	0.98	2.88	3.827 (5)	164
C11—H11*C*⋯Br1^ii^	0.96	2.94	3.874 (7)	165
C12—H12*B*⋯Br1^i^	0.97	2.97	3.855 (5)	152

Symmetry codes: (i) 
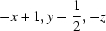
; (ii) 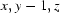.

**Fig. 1.**
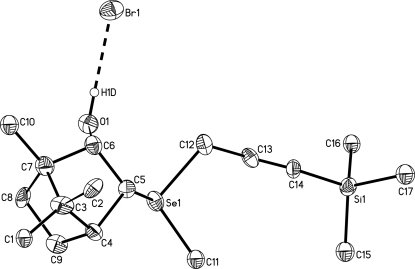
: View of the title compound, showing the labelling of the non-H atoms and 30% probability ellipsoids. H atoms have been omitted for clarity, except for H1D which is involved in hydrogen bonding.

**Fig. 2.**
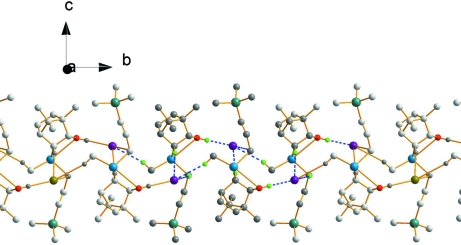
: A view of the onedimensional R-helical chains along the [010] axis. H atoms have been omitted for clarity, except for H1D, H5, H11c, and H12B which are involved in hydrogen bonding.

**Fig. 3.**
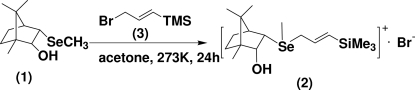
: Reaction scheme.

## supplementary crystallographic information

### Comment

Recently, an efficient asymmetric synthesis of cyclopropanes *via* camphor-derived sulfonium ylides was reported (Ye *et al.*, 2002). Thus, we expected that camphor-derived selenonium ylides could be used in the highly enantioselective synthesis of cyclopropanes, epoxides and aziridines. First, the camphor-derived selenide (1) was prepared from commercially available *D*-camphor according to a literature method (Reich *et al.*, 1975; Goodridge *et al.*, 1988, and Li *et al.*, 2005). Then compound (1) was reacted with (3-bromo-propenyl)-trimethylsilane (3) to give the selenonium salt (2). We performed the X-ray crystallographic analysis of (2) in order to elucidate the conformation and configuration.

The structural analysis shows that the selenonium ion of the title compound, (2) (Fig. 1), is not centrosymmetric and has no symmetry plane, showing the four chiral C atoms, C4, C5, C6, and C7, with the *R*, *R*, S, and S configuration preserved from the enatiomerically pure starting compound (1). The asymmetric unit contains one selenonium salt cation, and one bromide ion. In the crystal packing, the Br atom plays an important role, acting as a bridge linking neighboring molecules *via* C–H···Br and O–H···Br hydrogen bonds (O1—H1D···Br1, C5—H5···Br1^ii^, C11—H11*c*···Br1^i^, and C12—H12B···Br1^ii^; symmetry code i: *x*,-1 + *y*,*z*; ii: 1 - *x*,-1/2 + *y*, -*z*), forming a one dimensional *R*-helical chains-like structure along [010] axis (Fig. 2).

### Experimental

A solution of 4,7,7-trimethyl-2-methylselanyl-bicyclo[2.2.1]heptan-3-ol (1) (2.4 g, 9.7 mmol) and (3-bromo-propenyl)-trimethylsilane (3) (1.9 g, 9.7 mmol) in acetone (5 mL) was stirred at 273 K. The solid was collected and washed with ethyl ether to afford the selenonium salt (2) in 91% yield. Single crystals of (2) were obtained by slow evaporation from 10 mL of a methanolic solution containing 50 mg (2).

### Refinement

H atoms bonded to O atoms were located in a difference map and refined with distance restraints of O—H = 0.87 (10), and with *U*_iso_(H) = 1.2*U*_eq_(O). Other H atoms were positioned geometrically and refined using a riding model (including free rotation about the ethanol C—C bond), with C—H = 0.96–0.98 Å and with *U*_iso_(H) = 1.2(1.5 for methyl groups) times *U*_eq_(C).

### Crystal data

**Table:**

C_17_H_33_OSeSi^+^·Br^–^	*F*_000_ = 452
*M**_r_* = 440.39	*D*_x_ = 1.366 Mg m^−^^3^
Monoclinic, *P*2(1)	Mo *K*α radiation λ = 0.71073 Å
Hall symbol: P 2yb	Cell parameters from 895 reflections
*a* = 7.555 (2) Å	θ = 2.1–24.5º
*b* = 10.023 (2) Å	µ = 3.67 mm^−^^1^
*c* = 14.423 (3) Å	*T* = 291 (2) K
β = 101.29 (3)º	Bloc, colourless
*V* = 1071.0 (4) Å^3^	0.30 × 0.26 × 0.24 mm
*Z* = 2	

### Data collection

**Table:**

Bruker SMART Apex CCD diffractometer	3374 independent reflections
Radiation source: sealed tube	1732 reflections with *I* > 2σ(*I*)
Monochromator: graphite	*R*_int_ = 0.035
*T* = 291(2) K	θ_max_ = 26.0º
phi and ω scans	θ_min_ = 1.4º
Absorption correction: multi-scan(SADABS; Bruker, 2000)	*h* = −9→9
*T*_min_ = 0.35, *T*_max_ = 0.41	*k* = 0→12
4460 measured reflections	*l* = 0→17

### Refinement

**Table:**

Refinement on *F*^2^	Hydrogen site location: inferred from neighbouring sites
Least-squares matrix: full	H atoms treated by a mixture of independent and constrained refinement
*R*[*F*^2^ > 2σ(*F*^2^)] = 0.053	*w* = 1/[σ^2^(*F*_o_^2^) + (0.046*P*)^2^] where *P* = (*F*_o_^2^ + 2*F*_c_^2^)/3
*wR*(*F*^2^) = 0.102	(Δ/σ)_max_ < 0.001
*S* = 1.07	Δρ_max_ = 0.64 e Å^−^^3^
3374 reflections	Δρ_min_ = −0.74 e Å^−^^3^
170 parameters	Extinction correction: none
1 restraint	Absolute structure: Flack (1983), 1140 Friedel pairs
Primary atom site location: structure-invariant direct methods	Flack parameter: 0.01 (2)
Secondary atom site location: difference Fourier map	

### Special details

**Table:**

Geometry. All e.s.d.'s (except the e.s.d. in the dihedral angle between two l.s. planes) are estimated using the full covariance matrix. The cell e.s.d.'s are taken into account individually in the estimation of e.s.d.'s in distances, angles and torsion angles; correlations between e.s.d.'s in cell parameters are only used when they are defined by crystal symmetry. An approximate (isotropic) treatment of cell e.s.d.'s is used for estimating e.s.d.'s involving l.s. planes.
Refinement. Refinement of *F*^2^ against ALL reflections. The weighted *R*-factor *wR* and goodness of fit *S* are based on *F*^2^, conventional *R*-factors *R* are based on *F*, with *F* set to zero for negative *F*^2^. The threshold expression of *F*^2^ > σ(*F*^2^) is used only for calculating *R*-factors(gt) *etc*. and is not relevant to the choice of reflections for refinement. *R*-factors based on *F*^2^ are statistically about twice as large as those based on *F*, and *R*- factors based on ALL data will be even larger.

### Fractional atomic coordinates and isotropic or equivalent isotropic displacement parameters (Å^2^)

**Table:**

	*x*	*y*	*z*	*U*_iso_*/*U*_eq_	
Br1	0.32321 (7)	1.05851 (10)	0.09364 (5)	0.0584 (2)	
C1	0.6248 (8)	0.4347 (6)	0.3285 (4)	0.0404 (14)	
H1A	0.6335	0.3442	0.3079	0.061*	
H1B	0.5363	0.4400	0.3679	0.061*	
H1C	0.7399	0.4630	0.3637	0.061*	
C2	0.7389 (6)	0.5504 (10)	0.2095 (4)	0.0425 (13)	
H2A	0.8154	0.6083	0.2529	0.064*	
H2B	0.7085	0.5928	0.1488	0.064*	
H2C	0.8013	0.4683	0.2036	0.064*	
C3	0.5706 (8)	0.5219 (6)	0.2454 (5)	0.0498 (18)	
C4	0.4232 (11)	0.4559 (8)	0.1682 (5)	0.0445 (19)	
H4	0.4615	0.3724	0.1428	0.053*	
C5	0.3867 (6)	0.5695 (8)	0.0960 (4)	0.0361 (12)	
H5	0.4822	0.5710	0.0588	0.043*	
C6	0.3996 (9)	0.6917 (8)	0.1552 (5)	0.0398 (16)	
H6	0.4963	0.7496	0.1422	0.048*	
C7	0.4459 (9)	0.6398 (7)	0.2553 (4)	0.0473 (15)	
C8	0.2804 (6)	0.5707 (9)	0.2761 (4)	0.0406 (14)	
H8A	0.1744	0.6264	0.2578	0.049*	
H8B	0.2955	0.5499	0.3428	0.049*	
C9	0.2627 (8)	0.4411 (7)	0.2161 (5)	0.0460 (15)	
H9A	0.2728	0.3619	0.2555	0.055*	
H9B	0.1499	0.4384	0.1704	0.055*	
C10	0.5215 (9)	0.7500 (7)	0.3273 (5)	0.047	
H10A	0.6407	0.7741	0.3196	0.071*	
H10B	0.5262	0.7175	0.3903	0.071*	
H10C	0.4444	0.8269	0.3166	0.071*	
C11	0.2014 (9)	0.3828 (7)	−0.0510 (5)	0.043	
H11A	0.3020	0.3955	−0.0816	0.064*	
H11B	0.0971	0.3576	−0.0970	0.064*	
H11C	0.2294	0.3137	−0.0043	0.064*	
C12	0.2030 (8)	0.6908 (7)	−0.0809 (4)	0.0415 (15)	
H12A	0.1683	0.7780	−0.0614	0.050*	
H12B	0.3310	0.6926	−0.0820	0.050*	
C13	0.0925 (8)	0.6561 (7)	−0.1810 (4)	0.0430 (15)	
H13	−0.0322	0.6663	−0.1918	0.052*	
C14	0.1643 (8)	0.6141 (6)	−0.2502 (4)	0.0385 (13)	
H14	0.2893	0.6068	−0.2395	0.046*	
C15	−0.0572 (9)	0.3993 (7)	−0.3683 (5)	0.047	
H15A	−0.1696	0.4027	−0.3468	0.071*	
H15B	0.0262	0.3436	−0.3267	0.071*	
H15C	−0.0771	0.3631	−0.4311	0.071*	
C16	−0.1393 (8)	0.6949 (7)	−0.4057 (5)	0.041	
H16A	−0.2022	0.6764	−0.4689	0.061*	
H16B	−0.0837	0.7813	−0.4039	0.061*	
H16C	−0.2231	0.6937	−0.3636	0.061*	
C17	0.1943 (7)	0.5796 (7)	−0.4543 (4)	0.049	
H17A	0.2631	0.4987	−0.4522	0.073*	
H17B	0.2746	0.6536	−0.4372	0.073*	
H17C	0.1259	0.5926	−0.5171	0.073*	
O1	0.2297 (6)	0.7626 (5)	0.1376 (3)	0.0473 (11)	
H1D	0.248 (10)	0.845 (8)	0.125 (5)	0.057*	
Se1	0.15165 (6)	0.54982 (7)	0.01059 (4)	0.04094 (15)	
Si1	0.03564 (18)	0.5673 (2)	−0.36882 (11)	0.0395 (4)	

### Atomic displacement parameters (Å^2^)

**Table:**

	*U*^11^	*U*^22^	*U*^33^	*U*^12^	*U*^13^	*U*^23^
Br1	0.0503 (3)	0.0494 (4)	0.0775 (5)	−0.0034 (4)	0.0172 (3)	−0.0074 (5)
C1	0.043 (3)	0.043 (4)	0.040 (3)	0.000 (3)	0.020 (3)	0.000 (3)
C2	0.031 (2)	0.049 (3)	0.043 (3)	−0.012 (4)	−0.0060 (19)	−0.021 (5)
C3	0.038 (3)	0.046 (4)	0.053 (4)	−0.019 (3)	−0.020 (3)	0.003 (3)
C4	0.059 (4)	0.038 (4)	0.034 (4)	−0.005 (3)	0.004 (3)	0.008 (3)
C5	0.035 (2)	0.037 (3)	0.035 (3)	0.019 (3)	0.0062 (18)	0.005 (3)
C6	0.034 (3)	0.048 (4)	0.040 (4)	−0.005 (3)	0.013 (3)	0.003 (3)
C7	0.052 (3)	0.047 (3)	0.037 (4)	0.001 (3)	−0.003 (3)	0.006 (3)
C8	0.042 (2)	0.049 (4)	0.035 (3)	0.016 (3)	0.017 (2)	−0.009 (3)
C9	0.034 (3)	0.046 (4)	0.055 (4)	−0.004 (3)	0.004 (3)	0.005 (3)
C10	0.049	0.049	0.049	0.000	0.021	0.000
C11	0.044	0.044	0.044	0.000	0.018	0.000
C12	0.039 (3)	0.051 (4)	0.036 (3)	0.020 (3)	0.012 (3)	0.009 (3)
C13	0.045 (3)	0.048 (4)	0.033 (3)	0.005 (3)	0.002 (3)	0.014 (3)
C14	0.044 (3)	0.044 (3)	0.029 (3)	−0.004 (2)	0.012 (2)	−0.002 (3)
C15	0.049	0.049	0.049	0.000	0.021	0.000
C16	0.043	0.043	0.043	0.000	0.020	0.000
C17	0.050	0.050	0.050	0.000	0.020	0.000
O1	0.046 (2)	0.049 (3)	0.038 (2)	−0.010 (2)	−0.0126 (19)	0.003 (2)
Se1	0.0358 (2)	0.0516 (3)	0.0339 (3)	0.0028 (4)	0.00292 (19)	0.0086 (4)
Si1	0.0420 (7)	0.0402 (9)	0.0380 (8)	0.0077 (9)	0.0119 (6)	−0.0030 (10)

### Geometric parameters (Å, °)

**Table:**

Br1—O1	3.143 (5)	C10—H10B	0.9600
C1—C3	1.475 (9)	C10—H10C	0.9600
C1—H1A	0.9600	C11—Se1	1.965 (7)
C1—H1B	0.9600	C11—H11A	0.9600
C1—H1C	0.9600	C11—H11B	0.9600
C2—C3	1.492 (8)	C11—H11C	0.9600
C2—H2A	0.9600	C12—C13	1.560 (9)
C2—H2B	0.9600	C12—Se1	2.022 (6)
C2—H2C	0.9600	C12—H12A	0.9700
C3—C7	1.535 (9)	C12—H12B	0.9700
C3—C4	1.559 (9)	C13—C14	1.295 (8)
C4—C9	1.515 (10)	C13—H13	0.9300
C4—C5	1.531 (10)	C14—Si1	1.855 (6)
C4—H4	0.9800	C14—H14	0.9300
C5—C6	1.484 (10)	C15—Si1	1.825 (7)
C5—Se1	1.963 (5)	C15—H15A	0.9600
C5—H5	0.9800	C15—H15B	0.9600
C6—O1	1.446 (8)	C15—H15C	0.9600
C6—C7	1.510 (9)	C16—Si1	1.842 (7)
C6—H6	0.9800	C16—H16A	0.9600
C7—C8	1.510 (9)	C16—H16B	0.9600
C7—C10	1.546 (9)	C16—H16C	0.9600
C8—C9	1.552 (10)	C17—Si1	1.884 (5)
C8—H8A	0.9700	C17—H17A	0.9600
C8—H8B	0.9700	C17—H17B	0.9600
C9—H9A	0.9700	C17—H17C	0.9600
C9—H9B	0.9700	O1—H1D	0.87 (8)
C10—H10A	0.9600		
			
C3—C1—H1A	109.5	C7—C10—H10A	109.5
C3—C1—H1B	109.5	C7—C10—H10B	109.5
H1A—C1—H1B	109.5	H10A—C10—H10B	109.5
C3—C1—H1C	109.5	C7—C10—H10C	109.5
H1A—C1—H1C	109.5	H10A—C10—H10C	109.5
H1B—C1—H1C	109.5	H10B—C10—H10C	109.5
C3—C2—H2A	109.5	Se1—C11—H11A	109.5
C3—C2—H2B	109.5	Se1—C11—H11B	109.5
H2A—C2—H2B	109.5	H11A—C11—H11B	109.5
C3—C2—H2C	109.5	Se1—C11—H11C	109.5
H2A—C2—H2C	109.5	H11A—C11—H11C	109.5
H2B—C2—H2C	109.5	H11B—C11—H11C	109.5
C1—C3—C2	106.0 (5)	C13—C12—Se1	108.2 (4)
C1—C3—C7	117.3 (6)	C13—C12—H12A	110.1
C2—C3—C7	117.8 (6)	Se1—C12—H12A	110.1
C1—C3—C4	112.0 (5)	C13—C12—H12B	110.1
C2—C3—C4	111.8 (6)	Se1—C12—H12B	110.1
C7—C3—C4	91.6 (5)	H12A—C12—H12B	108.4
C9—C4—C5	109.2 (6)	C14—C13—C12	123.8 (5)
C9—C4—C3	103.9 (6)	C14—C13—H13	118.1
C5—C4—C3	100.3 (5)	C12—C13—H13	118.1
C9—C4—H4	114.0	C13—C14—Si1	124.7 (5)
C5—C4—H4	114.0	C13—C14—H14	117.6
C3—C4—H4	114.0	Si1—C14—H14	117.6
C6—C5—C4	103.8 (5)	Si1—C15—H15A	109.5
C6—C5—Se1	113.2 (4)	Si1—C15—H15B	109.5
C4—C5—Se1	111.9 (5)	H15A—C15—H15B	109.5
C6—C5—H5	109.3	Si1—C15—H15C	109.5
C4—C5—H5	109.3	H15A—C15—H15C	109.5
Se1—C5—H5	109.3	H15B—C15—H15C	109.5
O1—C6—C5	110.4 (5)	Si1—C16—H16A	109.5
O1—C6—C7	111.6 (5)	Si1—C16—H16B	109.5
C5—C6—C7	104.1 (6)	H16A—C16—H16B	109.5
O1—C6—H6	110.2	Si1—C16—H16C	109.5
C5—C6—H6	110.2	H16A—C16—H16C	109.5
C7—C6—H6	110.2	H16B—C16—H16C	109.5
C6—C7—C8	107.5 (5)	Si1—C17—H17A	109.5
C6—C7—C3	102.0 (5)	Si1—C17—H17B	109.5
C8—C7—C3	102.2 (5)	H17A—C17—H17B	109.5
C6—C7—C10	112.5 (6)	Si1—C17—H17C	109.5
C8—C7—C10	114.0 (6)	H17A—C17—H17C	109.5
C3—C7—C10	117.4 (5)	H17B—C17—H17C	109.5
C7—C8—C9	105.0 (5)	C6—O1—Br1	105.8 (4)
C7—C8—H8A	110.8	C6—O1—H1D	109 (5)
C9—C8—H8A	110.8	C5—Se1—C11	98.0 (3)
C7—C8—H8B	110.8	C5—Se1—C12	94.2 (3)
C9—C8—H8B	110.8	C11—Se1—C12	102.9 (3)
H8A—C8—H8B	108.8	C15—Si1—C16	112.8 (3)
C4—C9—C8	100.5 (5)	C15—Si1—C14	111.2 (3)
C4—C9—H9A	111.7	C16—Si1—C14	107.9 (3)
C8—C9—H9A	111.7	C15—Si1—C17	110.9 (3)
C4—C9—H9B	111.7	C16—Si1—C17	106.1 (3)
C8—C9—H9B	111.7	C14—Si1—C17	107.6 (3)
H9A—C9—H9B	109.4		

### Hydrogen-bond geometry (Å, °)

**Table:**

*D*—H···*A*	*D*—H	H···*A*	*D*···*A*	*D*—H···*A*
O1—H1D···Br1	0.87 (8)	2.28 (8)	3.143 (5)	175 (7)
C5—H5···Br1^i^	0.98	2.88	3.827 (5)	164
C11—H11C···Br1^ii^	0.96	2.94	3.874 (7)	165
C12—H12B···Br1^i^	0.97	2.97	3.855 (5)	152

Symmetry codes: (i) −*x*+1, *y*−1/2, −*z*; (ii) *x*, *y*−1, *z*.
